# Subcutaneous immunotherapy induces alterations in monocytes and dendritic cells homeostasis in allergic rhinitis patients

**DOI:** 10.1186/s13223-018-0271-8

**Published:** 2018-11-15

**Authors:** Letícia Sousa, Carmen Martín-Sierra, Celso Pereira, Graça Loureiro, Beatriz Tavares, Susana Pedreiro, António Martinho, Artur Paiva

**Affiliations:** 1 Stemlab, S.A, Biocant Park, Núcleo 4, Lote 2, Cantanhede, Portugal; 2 Flow Cytometry Unit, Clinical Pathology Service, Centro Hospitalar e Universitário de Coimbra, Praceta Prof. Mota Pinto, Ed. S. Jerónimo, 3° piso, 30001-301 Coimbra, Portugal; 3 Immunoallergology Department, Centro Hospitalar e Universitário de Coimbra, Coimbra, Portugal; 4 Portuguese Institute of Blood and Transplantation, Coimbra, Portugal; 5 CIMAGO-Center of Investigation on Environment Genetics and Oncobiology, Faculdade de Medicina da Universidade de Coimbra, Coimbra, Portugal

**Keywords:** Allergic rhinitis, Immunotherapy, Monocytes, Dendritic cells, IgE, TNFα

## Abstract

**Background:**

Specific subcutaneous immunotherapy (SCIT) can achieve long-term remission in patients with allergic rhinitis (AR) through complex and still unknown mechanisms. The aim of this study is to evaluate the effect of SCIT over CD16^+^ and CD16^−^ monocytes, myeloid (mDCs) and plasmacytoid dendritic cells (pDCs) in patients with AR, comparatively to pharmacological standard treatment (non-SIT).

**Methods:**

The relative frequency and absolute number of monocytes and DC subsets, the frequency of these cells producing TNFα after in vitro stimulation with *Dermatophagoides pteronyssinus* (Dpt) extract, and the expression levels of receptor-bound IgE or IgG were assessed by flow cytometry, in peripheral blood samples from 23 healthy individuals (HG) and 43 participants with AR mono-sensitized to Dpt; 10 with non-SIT treatment and 33 under SCIT, just before (SCIT-T0) and 4 h after administration (SCIT-T4). Moreover, IFNα mRNA expression was evaluated in purified pDCs, by qRT-PCR.

**Results:**

After SCIT administration we observed a strong decrease of circulating pDCs, although accompanied by higher levels of IFNα mRNA expression, and an increase of circulating CD16^+^ monocytes. AR participants under SCIT exhibited a higher expression of receptor-bound IgE in all cell populations that expressed the high affinity receptor for IgE (FcεRI) and a higher frequency of CD16^+^ monocytes producing TNFα. Conversely, we observed a decrease in the frequency of mDCs producing TNFα in AR under SCIT, similar to the observed in the control group.

**Conclusions:**

SCIT seems to induce numeric, phenotypic, and functional changes in circulating monocytes and dendritic cells, contributing at least in part to the well described immunological alterations induced by this type of immunotherapy.

## Background

Allergic rhinitis (AR) is an inflammation of the nasal mucous membranes mainly caused by immunoglobulin (Ig)E-mediated allergic reactions to otherwise innocuous inhaled aeroallergens, including pollen grains, mold spores, house dust mites and animal dander [1, 2]. AR has significant effects on the quality of life of patients, many of which are inadequately controlled. In fact, a study in Canada shows that many AR patients experience symptoms that could benefit from better treatment. A portion of the total disease burden is represented by common comorbid conditions such as asthma, sinusitis, nasal polyposis, and sleep apnea. But nasal congestion and runny nose have been reported as the most bothersome symptoms [3, 4]. AR and its symptoms are ultimately triggered by IgE-mediated immune responses against a foreign protein [1].

Antigen-presenting cells (APCs), especially dendritic cells (DCs), are known to play a fundamental role in the onset of allergic sensitization and in the repeated activation of Th2-mediated responses. Furthermore, DCs are thought to be a source of crucial Th2-attracting chemokines, including CCL17 (TARC or thymus and activation-regulated chemokine) and CCL22 (MDC or macrophage-derived chemokine) [5]. Moreover, FcεRI can also be found in the cell membrane of DCs and in a small subpopulation of monocytes. Therefore, the endocytosis of FcεRI-bound IgE by these cells could either result in degradation (IgE clearance) [6], or if the allergen is present in the complex, in the processing and peptide loading in nascent MHC class II molecules [7, 8]. Allergen specific immunotherapy (SIT) is thus far the only treatment option that changes the immunologic mechanism of allergy thereby modifying the natural course of the disease with the potential for long-term benefits as well as preventing sensitizations to new allergens. SIT consists in allergen administration to decrease sensitivity to the allergen; typically, through sublingual delivery (SLIT) or subcutaneous injections (SCIT).

The exact mechanisms underlying the clinical efficacy of SCIT treatment are not yet fully elucidated, although they are known to involve the induction of serological changes. These changes include the induction of a subclass of allergen-specific IgG antibodies with potent inhibitory activity against IgE that persists after treatment discontinuation, or changes in IgE serum levels, as well as changes in immune cells [9] by a widespread mechanism which includes the modulation of mast cells, basophils, T and B cells, and IgE production [9–15]. SCIT can also regulate immune responses by increasing the production of pro-inflammatory cytokines by DCs, such as IFNα and IL-6, and restoring their ability to respond to stimuli [9, 16].

The aim of this study was to evaluate the effect of SCIT with *Dermatophagoides pteronyssinus* (Dpt) extract over peripheral blood monocyte and DC subpopulations in Dpt-allergic AR participants, and compare it with conventional pharmacological treatment. For this purpose, we determined by flow cytometry the frequency of monocyte subpopulations and myeloid DCs (mDCs) producing TNFα after in vitro stimulation with Dpt, as well as the expression levels of receptor-bound IgE and IgG to their specific receptors, in CD16^+^ and CD16^−^ monocytes, mDCs, and plasmacytoid dendritic cells (pDCs) prior to and 4 h after allergen injection. Since stimulation of monocytes significantly downregulates CD16 expression [17–19], intermediate and non-classical monocytes became very difficult to identify after the stimulation procedure. In the case of Dpt stimulation, the effects observed over CD16 expression were lower than the previously described, but it remained difficult to properly identify intermediate and non-classical monocytes. Therefore, monocyte subpopulations were divided into CD16^+^ (including non-classical and intermediate) and CD16^−^ (classical) monocytes. Moreover, we evaluated IFNα mRNA expression in purified pDCs by qRT-PCR.

## Methods

### Participants

This study included a control group of 23 individuals (11 women and 12 men, with an average of 28 ± 9 years of age) without medical history of allergic disease, without any treatment with immunomodulatory drugs, and free from autoimmune diseases and active infection (HG); a group of 10 participants (7 women and 3 men, with an average of 27 ± 7 years of age) with respiratory allergy (rhinitis, with or without allergic asthma) to house dust mite *Dermatophagoides pteronyssinus* (Dpt), under conventional pharmacological treatment and that had never been submitted in the past to sublingual or subcutaneous immunotherapy (non-SIT); and 33 participants (14 women and 19 men, with an average of 31 ± 11 years of age) with respiratory allergy, rhinitis and allergic controlled asthma to Dpt, submitted to maintenance SCIT (polymerized glutaraldehyde Dpt extract, Bial-Aristegui, Bilbao, Spain) for at least 1 year (with a mean treatment period of 28 ± 13 months) (SCIT). The SCIT group was further subdivided according to injection time, to compare treatment efficacy: immediately before SCIT administration (SCIT-T0) and 4 hours after treatment administration (SCIT-T4). Inclusion criteria for this group of allergic participants included absence of active infection and inflammation and/or other concomitant clinical disorders. At the moment of the treatment implementation, the diagnosis of persistent moderate/severe rhinitis (ARIA Classification), and the presence of concomitant mild persistent controlled asthma (GINA Classification) were not exclusion criteria [20, 21].

All AR participants were clinically evaluated according to symptoms, positive skin prick tests, and serum specific IgE assays to Dpt (ImmunoCAP Specific IgE, Thermoscientific, Uppsala, Sweeden).

Skin prick tests to a panel of aeroallergens (including house dust and storage mites, moulds, pollens from *Poaceae*, weeds and trees representative of the region, cat and dog dander by BialAristegui, Bilbao, Spain), as well as histamine hydrochloride (10 mg/ml) and saline, as positive and negative controls, respectively (BialAristegui, Bilbao, Spain), were performed in all the participants. Lancets with 1 mm were used for skin pricking (Stallergenes, Antony France). The mean of the longest and the midpoint orthogonal diameters (mean diameter) of wheal size were considered for analysis and a wheal diameter ≥ 3 mm greater than that induced by the negative control was regarded as positive [22].

In all AR participants, specific nasal challenge test had demonstrated the etiology, according to standard procedures [23]. Nasal provocation tests (NPT) were performed first with saline solution applying two consecutives puffs (total volume of 0.10 ml) to the inferior nasal turbinate of the less congested nostril, using a nasal applicator spraying. Patients were asked to perform apnea during the allergen spraying. If negative, 10 min later, they were submitted to the previous protocol using a *Dermatophagoides pteronyssinus* extract (0.23 µg of Der p 1, BialAristegui, Bilbao, Spain). They were evaluated for the following 10 min according to the total nasal symptom score (attending sneezing, nasal pruritus, rhinorrhea, nasal obstruction, and ocular symptoms) and by the measure of the peak nasal inspiratory flow (PNIF). The test was considered positive if the patients achieved subjective and objective measures: increase of ≥ 5 points in the total nasal symptom score and a flow decrease of ≥ 40% of PNIF related to basal ratios [23, 24].

In the SCIT group, a second nasal challenge test was performed 1 month before the study. The test was negative for 25 participants, 5 participants showed response to an allergen concentration 100-fold higher, and the other 3 participants to a concentration 1000-fold higher than the initial dose previously used to confirm the diagnosis.

At the time of the study, the active group of participants was completely free of symptoms and no rescue medication or nasal anti-inflammatory therapy was needed. In the allergic control group (non-SIT), treatment was implemented according to the recommended guidelines (oral systemic anti-histamines, nasal and bronchial corticotherapy and bronchodilators for patients with asthma). Of note, all the analyses were performed during a period of clinical stabilization in both AR groups.

The clinical and laboratorial characteristics of all participants included in this study are presented in Table 1.

**Table 1:** Clinical and laboratorial characteristics of the individuals included in the study

	HG (n = 23)	Non-SIT (n = 10)	SCIT (n = 33)
Age (years)			
Mean ± SD	28 ± 9	27 ± 7	31 ± 11
Range	18–50	19–39	17–61
Gender			
Male	52.2% (n = 12)	30% (n = 3)	57.6% (n = 19)
Female	47.8% (n = 11)	70% (n = 7)	42.4% (n = 14)
Clinical parameters			
Time from disease onset (years)	NA	10 ± 7	12 ± 8
Time under SCIT (months)	NA	NA	28 ± 13
Presence of Asthma	NA	40% (n = 4)	43% (n = 13)
Laboratorial parameters			
Total serum IgE (t-IgE) (kU/L)	NA	295 ± 231	514 ± 787
Serum specific IgE (s-IgE) (kU_A_/L)	NA	48 ± 27	45 ± 35

The results were given by mean ± standard deviation (SD)

*NA* not applicable

### Ethical standards

All the participants with an allergic disease were selected from the Immunoallergology outpatient Department from Centro Hospitalar e Universitário de Coimbra.

The study protocol was approved by the Ethical Committee from Coimbra University Hospital (document number HUC-49-10) and all participants gave their signed informed consent.

### TNFα expression in monocyte subpopulations and mDCs after in vitro stimulation with Dpt

Peripheral blood samples were collected from allergic participants and healthy individuals into lithium heparin (Becton–Dickinson Biosciences, BD, San Jose, CA, USA) and K3-EDTA tubes (BD). Duplicates of 500 μL of lithium heparin anticoagulated peripheral blood were diluted 1/2 (vol/vol) in RPMI-1640 medium (Roswell Park Memorial Institute, Gibco, Carlsbad, USA) and Brefeldin A (Sigma-Aldrich, St. Louis, USA) was added to each tube leading to a final concentration of 10 μg/ml in each tube, to prevent the release of cytokines outside the cells. One of the tubes was stimulated with an extract of the allergic protein Der p 1, from Dpt (23 μg/ml Der p 1, Bial-Aristegui^®^, Bilbao, Spain) and the unstimulated sample was used as negative control. Both tubes were incubated for 6 h at 37 °C in a humidified atmosphere with 5% of CO_2_.

Each cultured sample was aliquoted (300 µl) into one tube and stained with CD16-Pacific Blue (PB, clone 3G8, Biolegend, San Diego, USA), HLA-DR-Fluorescein Isothiocyanate (FITC, clone Immu-357, Beckman Coulter, Marseille, France), CD11c-Peridinin-chlorophyll protein cyanine 5.5 (PerCP-Cy5.5, clone Bu15, Biolegend), CD45-Pacific Orange (PO, clone HI30, Life Technologies, New York, USA), CD14-Allophycocyanin-hilite 7 (APC-H7, clone MφP9, BD) and CD33-Allophycocyanin (APC, clone P67.6, BD) for 15 min in the dark at room temperature (RT). All samples were subjected to a permeabilization and staining protocols for the analysis of intracellular expression of TNFα-Phycoerythrin (PE, clone MAb11, BD) in mDCs and monocyte subsets. Samples were centrifuged twice (5 min at 540 g) in 2 ml of phosphate-buffered saline (PBS), resuspended in 0.5 ml of PBS and stored at 4 °C before acquisition.

### IgE and IgG bound to their specific receptors on the membrane of monocytes and dendritic cells

Each sample was aliquoted (300 µl) into one tube and stained with anti-IgE-PE (clone BE5, EXBIO Praha, Vestec, Czech Republic), anti-IgG-FITC (clone G18-145, BD), anti-HLA-DR- PerCP-Cy5.5 (clone G46-6, BD), anti-CD123-APC (clone AC145, Miltenyi Biotec; Bergisch, Gladbach, Germany), anti-CD16-PB (clone 3G8, Biolegend), anti-CD14-APC-H7 (clone MφP9, BD) and anti-CD45-krome orange (clone J.33, Beckman Coulter) for 15 min in the dark at RT. Then samples were incubated with 2 ml of FACS Lysing solution (BD) for 10 min in the dark at RT and centrifuged for 5 min at 540 g. The supernatant was discarded and the cell pellet was washed twice in 2 ml of PBS with centrifugation of 5 min at 540 g, resuspended in 0.5 ml of PBS, and stored at 4 °C before acquisition.

### Flow cytometry data acquisition and analysis

Data acquisition was performed in a FACSCanto™ II flow cytometer (BD) and analyzed with Infinicyt™ 1.4 software (Cytognos SL, Salamanca, Spain).

**Fig. 1:**
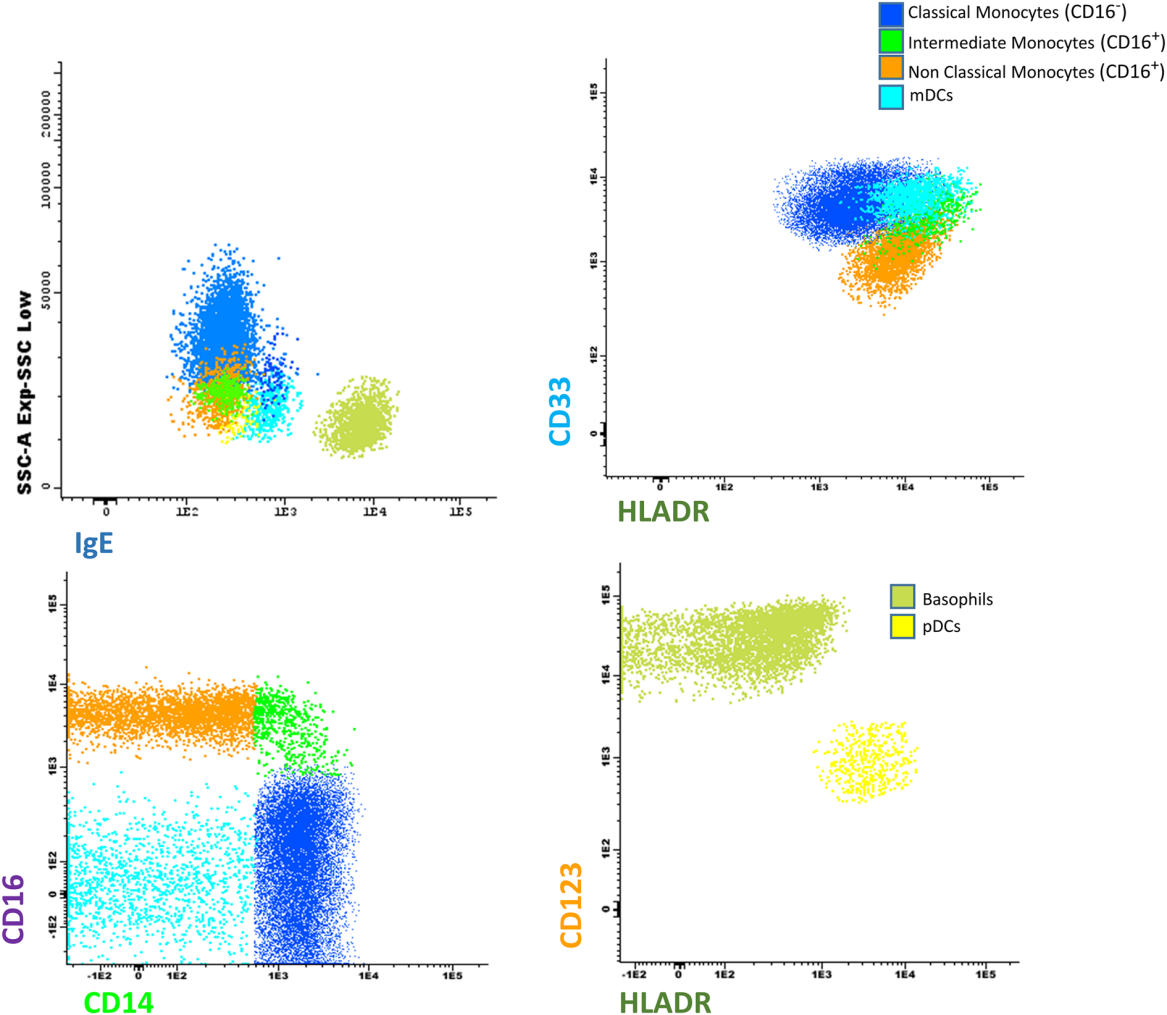
Phenotypic characteristics of peripheral blood classical (CD16^−^), intermediate and non-classical monocytes (CD16^+^), mDCs, and pDCs. Bivariate dot plot histograms illustrating the phenotypic strategy for the identification of the different monocyte subpopulations, pDCs and mDCs from peripheral blood. Classical monocytes (CD16^−^, blue events) express CD14 in the absence of CD16, they also show high reactivity for CD33, and HLA-DR; intermediate monocytes (CD16^+^, green events) are characterized as CD14-positive displaying an increasing positivity to CD16, together with positivity for CD33, and HLA-DR; non-classical monocytes (CD16^+^, orange events) are CD16-positive with a decreasing expression of CD14, presenting the lowest CD33 expression among monocytes subpopulations; mDCs (light blue events) are phenotypically characterized as negative for CD14, CD16, and they present lower SSC properties and higher expression of HLA-DR, CD33 and IgE bound to high affinity FcεRI compared to monocytes; pDCs are characterized by the high levels of CD123, but lower than basophils and high levels of HLA-DR expression

mDCs were characterized by the high expression of CD33, HLA-DR and IgE bound to its receptor, lower SSC light dispersion properties, lower CD45 expression compared to monocytes, and absence of CD16 and CD14 expression (Fig. 1); pDCs were characterized by the high levels of CD123 and HLA-DR expression, and absence of CD33 expression (Fig. 1); CD16^+^ monocytes were characterized by the bright CD45 expression, as well as expression of CD16; finally, classical or CD16^−^ monocytes were characterized by high levels of CD14 in the absence of CD16, together with high expression of CD33 and HLA-DR (Fig. 1).

### Cell sorting and purification of pDCs

6 ml of peripheral blood collected in K3-EDTA were lysed with ammonium chloride (NH_4_CL, Sigma-Aldrich) for 20 min horizontally at RT. After incubation, samples were centrifuged at 540 g for 5 min, the supernatant was discarded and the cell pellet was incubated with HLA-DR-FITC (clone Immu-357, Beckman Coulter), CD33-PE (clone P67.6, BD), CD14-PerCP-Cy5.5 (clone M5E2, BD Pharmingen, San Diego, USA), CD16-PE-Cy7 (clone 3G8, BD Pharmingen) and CD123-APC (clone 7G3, BD) for 20 min in the dark, at RT. After washing twice with PBS (540 g, 5 min), pDCs were purified in a FACSAria II cell sorter (BD), based on their positivity to HLA-DR, strong positivity for CD123, and negativity for CD14 and CD16. Purified cells were stored at − 80 °C. The purity of the sorted cells was ≥ 95%.

### Evaluation of IFNα mRNA expression by qRT-PCR

Cell suspensions were centrifuged for 5 min at 300 g and the pellet was resuspended in 350 µl of RLT Lysis Buffer (Qiagen, Hilden, Germany). Total RNA was extracted and purified in QIAcube (Qiagen) with RNeasy™Micro Kit (Qiagen) according to manufacturer’s instructions. Total RNA was eluted in 50 µl of RNAse-free water. RNA quantity and integrity were evaluated with a 6000 Nano Chip™ Kit in an Agilent 2100 bioanalyzer (Agilent, Walbronn, Germany). Reverse transcription was performed with SuperScript™ III First-Strand Synthesis SuperMix for qRT-PCR (Invitrogen, Carlsbad, CA, USA) according to supplier’s instructions, and relative quantification of gene expression was performed in a LightCycler™ 480 II (Roche Diagnostics, Rotkreuz, Switzerland) by a real time (qRT)-PCR reaction. To select optimal housekeeping genes, normalization of gene expression was executed with geNorm Housekeeping Gene Selection Human Kit (Primer Design, Southampton, UK) and geNorm™ software (Center for Medical Genetics, Ghent University Hospital, Ghent, Belgium). qRT-PCR was done with QuantiTect SYBR Green PCR Kit Gene expression, using optimized primers for IFNα and endogenous controls as beta-actin (ACTB) and glyceraldehyde 3-phosphate dehydrogenase (GAPDH) (Qiagen), according to the manufacturer’s instructions.

### Statistical analysis

The statistical analyses were performed using the Statistical Package for the Social Sciences v. 20 (SPSS Inc., Chicago, USA) software. The nonparametric Mann–Whitney U test for independent variables, the parametric Student’s t test, for comparing differences between related groups, and the Spearman’s rank correlation, to detect correlations between different parameters, were performed and differences were considered statistically significant when p < 0.05.

## Results

### Relative and absolute quantification of peripheral blood monocytes and DC subpopulations

After 4 h of SCIT administration we observed a strong depletion of pDCs (SCIT-T4), though the percentage and absolute values of these cells were higher in AR participants independently of the therapeutic protocol (non-SIT and SCIT-T0), when compared with the control group (HG).

On the other hand, after 4 h of SCIT administration, the mean percentage and absolute value (number of cell/µl) of CD16^+^ monocytes increased, despite those values being significantly lower in AR participants independently of the therapeutic protocol (non-SIT and SCIT-T0), compared with the HG.

mDCs were decreased in AR participants under conventional pharmacological treatment (non-SIT), whereas SCIT seemed to increase the percentage and absolute value of these cells to similar levels observed in the HG (Table 2).

**Table 2:** Frequency among total leukocytes (%, percentage) and absolute value (number of cell/µL) of peripheral blood CD16^−^ monocytes, CD16^+^ monocytes, myeloid (m)DCs and plasmacytoid (p)DCs

	HG	Non-SIT	SCIT-T0	SCIT-T4
CD16^−^ monocytes				
%	2.93 ± 1.57	5.12 ± 1.24	4.08 ± 1.14	3.40 ± 1.01
Absolute value	228.64 ± 132.41	419.36 ± 156.73	307.01 ± 93.81	260.20 ± 99.59
CD16^+^ monocytes				
%	0.32 ± 0.20	0.13 ± 0.08^a^	0.19 ± 0.15^b^	0.33 ± 0.15^ef^
Absolute value	24.47 ± 16.00	10.92 ± 6.90^a^	14.29 ± 12.14^b^	25.18 ± 12.99^ef^
mDCs				
%	0.21 ± 0.08	0.13 ± 0.07^a^	0.20 ± 0.10	0.19 ± 0.10
Absolute value	16.21 ± 7.12	9.77 ± 5.65^a^	14.87 ± 8.02	14.75 ± 10.02
pDCs				
%	0.13 ± 0.10	0.24 ± 0.10	0.28 ± 0.18^b^	0.05 ± 0.03^cef^
Absolute value	9.88 ± 6.52	19.52 ± 10.23^a^	21.58 ± 14.36^b^	3.55 ± 2.08^cef^

p < 0.05 for ^a^ Control vs Non-SIT; ^b^ Control vs SCIT-T0; ^c^ Control vs SCIT-T4; ^d^ Non-SIT vs SCIT-T0; ^e^ Non-SIT vs SCIT-T4; ^f^ SCIT-T0 vs SCIT-T4. The results were given by mean ± standard deviation

### Expression of receptor-bound IgE and IgG

**Fig. 2:**
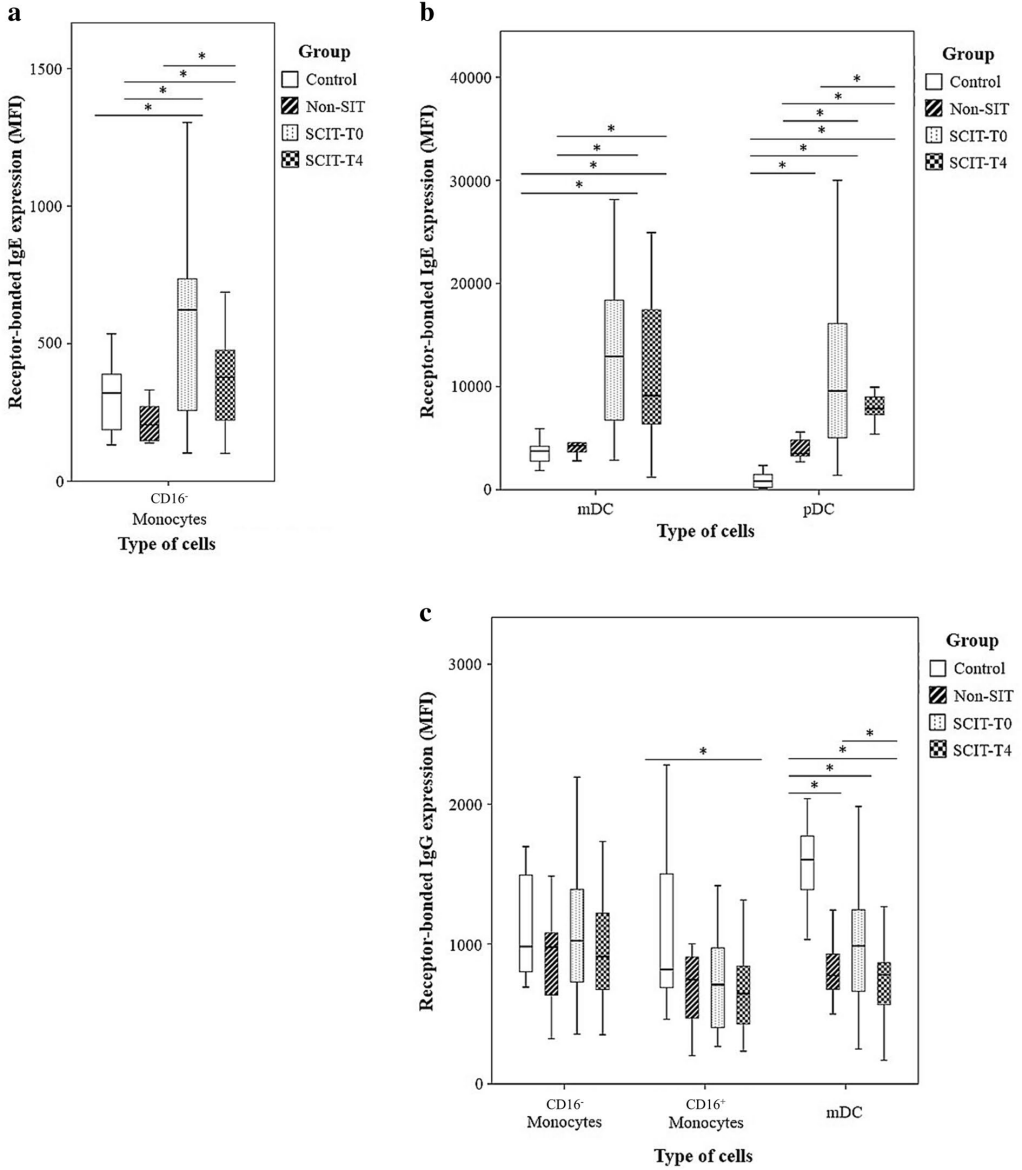
Receptor-bound IgE and IgG in monocyte and DC subpopulations. **a**, **b** Amount of receptor-bound IgE expressed per cell (MFI), measured in the control group, non-SIT group and in SCIT group, immediately before treatment (T0) or 4 h later (T4), among CD16^−^ monocytes (**a**), and myeloid (mDCs) and plasmacytoid (pDCs) dendritic cells (**b**). **c** Amount of receptor-bound IgG expressed per cell (MFI) among CD16^−^ and CD16^+^ monocytes and mDCs. Mann–Whitney U test was used to compare control, non-SIT and SCIT-T0/T4 groups. Student’s t test was used to compare SCIT-T0 versus the SCIT-T4 group. The results were given by median with interquartile range. Statistical significant differences were considered when p < 0.05; *between the groups indicated in the figure

The expression of receptor-bound IgE per cell in the SCIT group was significantly higher compared to the control and non-SIT groups, in all the studied cell populations expressing the FcεRI receptor (Fig. 2a, b).

The expression levels of receptor-bound IgG on mDCs and CD16^+^ monocytes were significantly lower in AR participants, independently of the therapeutic protocol, when compared with the control group (Fig. 2c).

### Frequency of monocyte subpopulations and mDCs producing TNFα following in vitro stimulation with Dpt

**Fig. 3:**
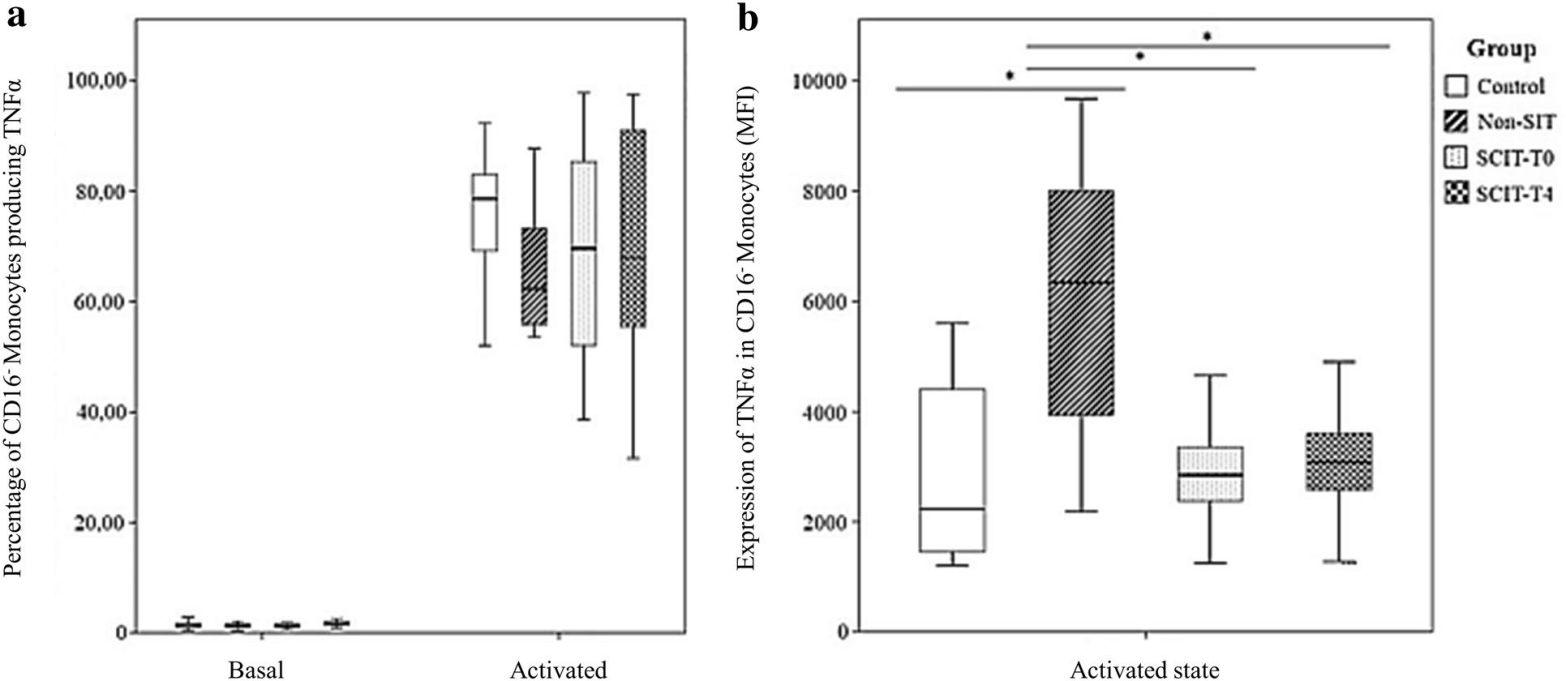
Frequency of TNFα producing cells (**a**) and relative amount of TNFα produced per cell (MFI) (**b**) among CD16^−^ monocytes, following in vitro stimulation with *Dermatophagoides pteronyssinus* (activated state) or without stimulation (basal state), in the control group, non-SIT group and SCIT group, immediately before treatment (T0) or 4 h after (T4). Mann–Whitney U test was used to compare control, non-SIT and SCIT-T0/T4 groups. Student’s t test was used to compare SCIT-T0 versus the SCIT-T4 group. The results were given by median with interquartile range. Statistical significant differences were considered when p < 0.05; *between the groups indicated in the figure

**Fig. 4:**
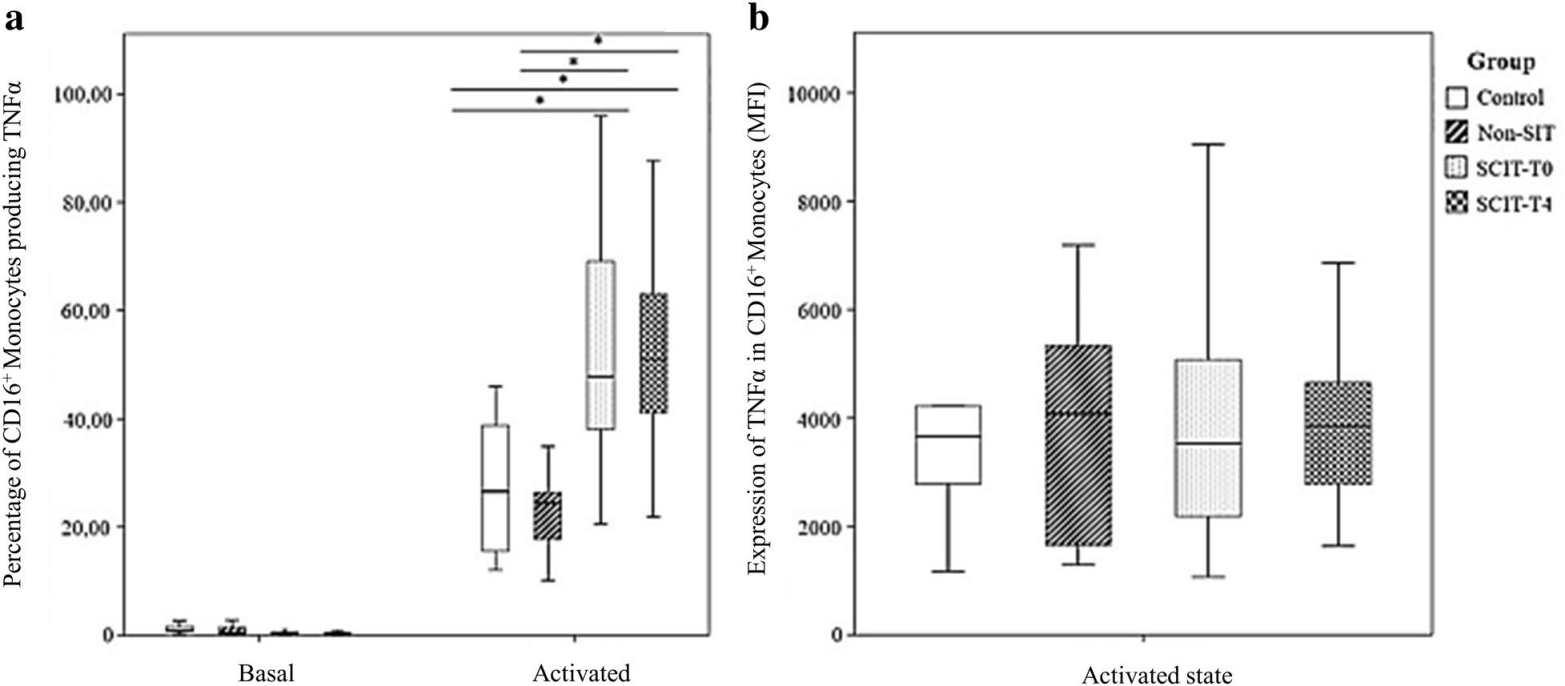
Frequency of TNFα producing cells (**a**) and relative amount of TNFα produced per cell (MFI) (**b**) among CD16^+^ monocytes, following in vitro stimulation with *Dermatophagoides pteronyssinus* (activated state) or without stimulation (basal state), in the control group, non-SIT group and SCIT group, immediately before treatment (T0) or 4 h after (T4). Mann–Whitney U test was used to compare control, non-SIT and SCIT-T0/T4 groups. Student’s t test was used to compare SCIT-T0 versus the SCIT-T4 group. The results were given by median with interquartile range. Statistical significant differences were considered when p < 0.05; *between the groups indicated in the figure

**Fig. 5:**
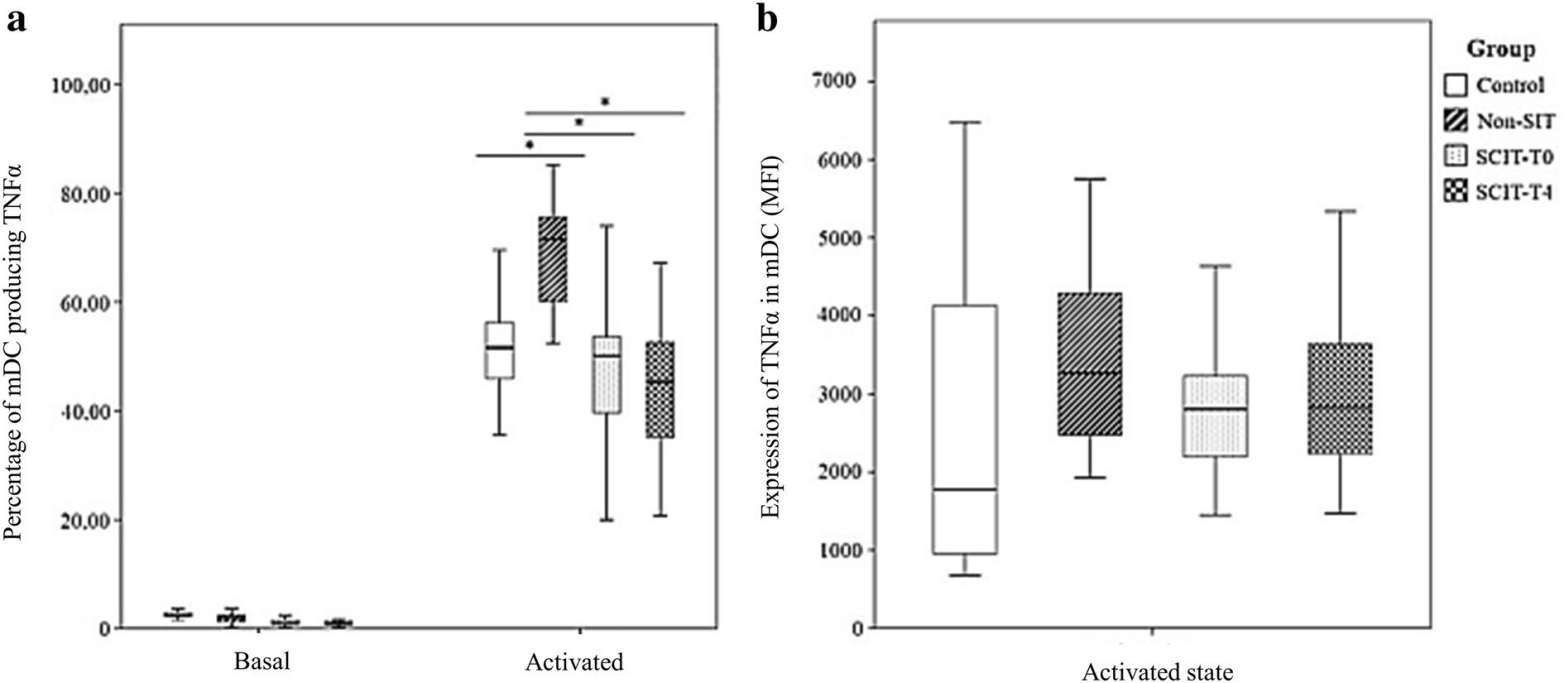
Frequency of TNFα producing myeloid dendritic cells (mDCs) (**a**) and amount of TNFα produced per cell (MFI) (**b**), with (activated state) or without activation (basal state) with *Dermatophagoides pteronyssinus*, in the control group, non-SIT group and SCIT group, immediately before treatment (T0) or 4 h after (T4). Mann–Whitney U test was used to compare control, non-SIT and SCIT-T0/T4 groups. Student’s t test was used to compare SCIT-T0 versus the SCIT-T4 group. The results were given by median with interquartile range. Statistical significant differences were considered when p < 0.05; *between the groups indicated in the figure

The frequency of CD16^−^ monocytes producing TNFα did not differ between the studied groups. However, the amount of TNFα produced per cell was clearly higher in non-SIT group when compared with the other groups (Fig. 3b). Conversely, no differences were observed in the amount of TNFα produced per cell in activated CD16^+^ monocytes and mDCs between the studied groups (Figs. 4b and 5b), but the frequency of CD16^+^ monocytes producing TNFα was markedly higher in the SCIT-T0 and SCIT-T4 groups (Fig. 4a). Finally, the frequency of mDCs producing TNFα was higher in the non-SIT group in comparison with the HG and the SCIT groups (SCIT-T0 and SCIT-T4) (Fig. 5a).

### Correlation between receptor-bound IgE expression and time under SCIT

**Fig. 6:**
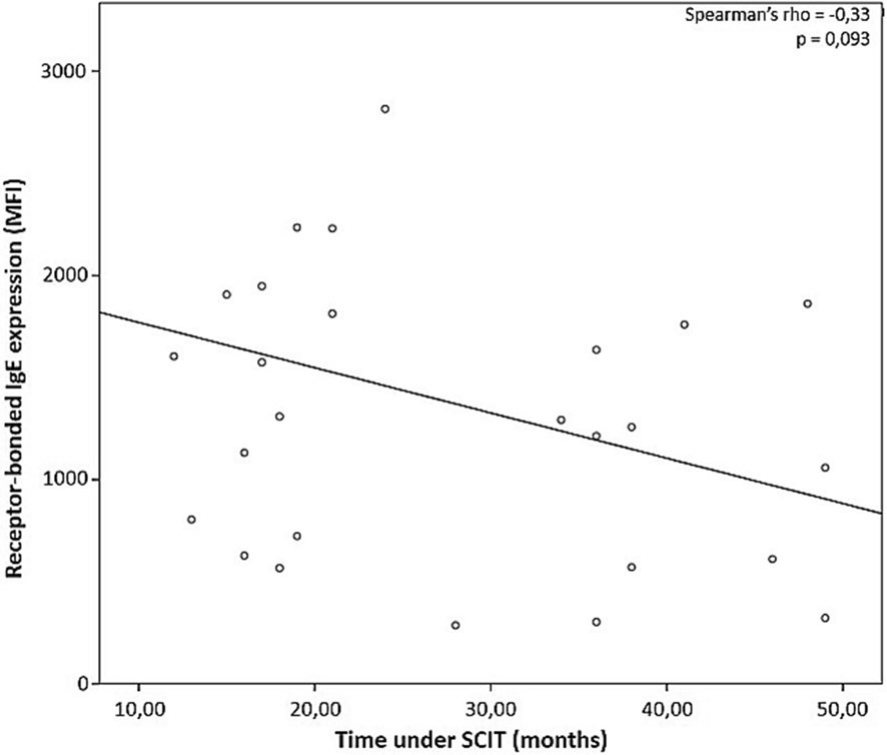
Correlation between receptor-bound IgE expression (MFI) and time under SCIT. Negative correlation between receptor-bound IgE expression (MFI) in myeloid dendritic cells (mDCs) and time under SCIT (months) (rho = − 0.33), as assessed by Spearman’s rank correlation, with p = 0.093

Receptor-bound IgE expression in mDCs showed a tendency to decrease over time under SCIT treatment, almost reaching statistical significance (p = 0.093, Fig. 6). No correlations were observed for receptor-bound IgE or IgG expression over time of SCIT treatment for all the other cell subpopulations under study (data not shown).

### IFNα mRNA expression in purified pDCs

**Fig. 7:**
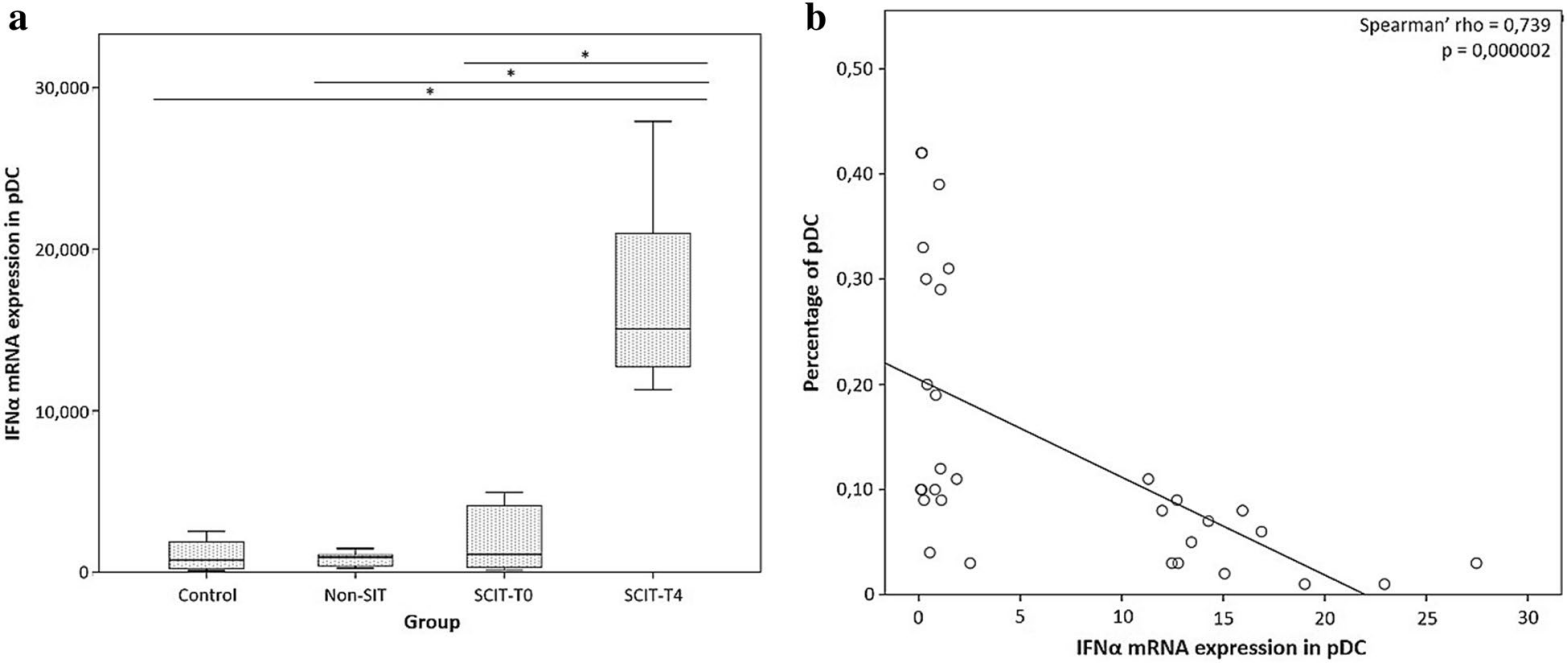
IFNα mRNA expression in plasmacytoid dendritic cells (pDCs). **a** Semi-quantitative analysis of IFNα mRNA expression among purified pDCs from the control group, non-SIT group and SCIT group, immediately before treatment (T0) and 4 h after (T4). **b** Negative correlation between the frequency of pDCs among total leukocytes (%) in SCIT-T4 group and IFNα mRNA expression in purified pDCs (rho = − 0.33), as assessed by Spearman’s rank correlation, with statistical significance (p = 0.000002)

IFNα mRNA expression among pDCs was significantly higher in SCIT-T4 when compared to the other groups (Fig. 7a). In addition, we observed a negative correlation between IFNα mRNA expression in pDCs and the frequency of pDCs among total leukocytes in the same group (Fig. 7b).

## Discussion

SIT is the only strategy that allows for immediate and long-term clinical efficiency in the restraint of the reactivity to the allergen, as well as the only one having a preventive effect against the development of new allergies [20]. However, not much is known about the exact underlying mechanism responsible for the therapeutic response to SIT. Growing evidence points to a modulation of T cell differentiation, a process mainly regulated by APCs. Nonetheless, the impact of SIT on APC subpopulations of allergic patients remains unknown, along with the mechanisms through which those changes are achieved. To address this issue, in the present study we compared the effect of the pharmacological treatment (non-SIT) and subcutaneous immunotherapy (SCIT: before SCIT administration, SCIT-T0; and 4 h after SCIT injection, SCIT-T4) in participants with AR. More specifically, we studied the effects on the number, phenotype and the function of peripheral blood monocytes, mDCs and pDCs, compared to a control group of healthy individuals (HG).

Due to their location in the skin, respiratory tract, and mucous membranes, APCs, and DCs in particular, are one of the first cells to enter in contact with allergens including allergenic substances administrated in SIT [20]. Consequently, these cells are involved in the initial signal of allergen sensitization and can control T cell differentiation through their ability to prime T cells into activated pro-inflammatory effector cell subsets or suppressive anti-inflammatory regulatory T cell subtypes [20, 21]. Additionally, DCs from participants with AR have an increased number in the nasal mucosa and selectively activate Th2 cell responses, promoting the progression of the disease [21].

Regarding the percentage and absolute number of cells, we observed that non-SIT treatment resulted in an increase in the percentage and absolute number of pDCs in peripheral blood. Likewise, a similar increase was found in the SCIT-T0 group. However, the percentage and absolute number of peripheral blood pDCs, 4 h after Dpt administration, were noticeably decreased. This rapid effect over pDCs had been previously reported by other authors [25–28]. Based on previous studies [28–32], we hypothesized that the observed decrease after SCIT was due to a specific migration of pDCs to the site of allergen contact, after DC activation. However, it is also possible that circulating pDCs were recruited to draining lymph nodes.

In addition to the decrease in circulating pDCs, the expression of IFNα mRNA by these cells was augmented 4 h after SCIT, in comparison with the control group, which is in line with previous studies showing that IFNα production by pDCs is severely impaired in allergic patients [9, 33], but can be restored through SCIT treatment involving a complex and still unknown mechanism [9]. The reduction of pDCs’ ability to produce IFNα in allergic subjects is likely to be due to a counter-regulation of the high affinity IgE receptor (FcεRI) signaling and antiviral responses in human pDCs [9, 33]. Thus, the increase of IFNα production might be indicative of a down-regulation of the FcεRI pathway.

Furthermore, while type I interferons are widely accepted as extremely potent antiviral cytokines, they also have a role in the counter-regulation of Th2 and Th17 cell responses [34]. Hence, up-regulation of IFNα expression by pDCs could aid in the skewing of T cell responses from Th2 to Th1, in allergic patients.

Therefore, the observed decrease in circulating pDCs, as well as the increase of IFNα mRNA expression, and the clear negative correlation between these two parameters, suggest an influx of pDCs that have the ability to promote Th1 cell polarization into the allergen-exposed site.

Conversely, the number and percentage of circulating mDCs were undisturbed in the two SCIT groups, compared to the HG. Although some studies had described that mDCs decline in number following allergen challenge [35], the aforementioned study by Dreschler et al., which focused specifically on subcutaneous SIT treatment in allergic patients, reported that the number of mDCs in peripheral blood was unchanged [28]. It has been demonstrated that distinct monocyte subpopulations exhibit distinct migratory potential in response to inflammation [36]; besides, some previous studies demonstrated that allergic patients display different profiles of peripheral blood monocyte subtypes, comparatively to healthy individuals, and that allergen challenge differentially affects the numbers of each subset, seemingly without affecting the total number of monocytes [37, 38]. We also observed that the percentage and absolute number of peripheral blood CD16^+^ monocytes were reduced in non-SIT and SCIT-T0 groups, comparatively to the HG. However, 4 h after the subcutaneous administration of Dpt extract, the number and the frequency of this monocyte subpopulation in the SCIT-T4 group, increased and reached similar values to those observed in the HG. The increase of circulating CD16^+^ monocytes after SCIT administration suggests a higher degree of differentiation of CD16^**−**^ monocytes into CD16^+^ monocytes. Furthermore, the percentage of this cell subset is often augmented in situ during inflammatory diseases [39] and DCs differentiated from CD16^+^ monocytes have been shown to reach more advanced levels of maturation and have a greater ability to induce IL-4 production by T cells than CD16^−^ monocyte-derived DCs [40].

Regarding the percentage and absolute number of peripheral blood CD16^−^ monocytes, we did not find significant differences between the studied groups, though they appeared to be increased (without reaching statistical significance) in non-SIT and SCIT-T0 groups, compared to the HG and SCIT-T4 groups, which is consistent with the proposed theory of differential monocyte migration.

In patients with allergic diseases, APCs in the skin or oral and nasal mucosa display high amounts of surface FcεRI, and FcεRI-bound IgE [41]. One of the most well established effect of SCIT is to induce an initial rise in allergen specific IgE, without significant illness effects, immediately followed by a decline to pre-treatment levels [9–12]. Furthermore, the amount of receptor bound-IgE in basophils, eosinophils, DCs and monocytes has been shown to be correlated with the levels of serum IgE, due to the FcεRI up-regulation triggered by the binding of IgE to the high affinity receptor [42–45]. Moreover, SIT is also associated with an increase in IgG levels, particularly allergen-specific IgG1 and IgG4 [14, 15]. This increase in the production of “blocking” antibodies, has been suggested to be the responsible for IgE neutralization during SIT through direct competition with IgE for allergen binding [11, 13–15]. However, the topic is still under debate; firstly, because the increase in the quantity of IgG seems to occur after the first signs of therapeutic efficacy, rather than before [12]; secondly, because mucosal DCs and mast cells come directly in contact with the allergen before IgG can exert their blocking activity [12]; and, thirdly, because of the poor or non-existing correlations between IgG levels and clinical benefit [10–12, 14, 43, 46]. Therefore, we studied the amount of receptor-bound IgE per cell, as well as the receptor-bound IgG expression in different subpopulations of APCs. In the SCIT-T0 and SCIT-T4 groups there was a significantly higher amount of receptor-bound IgE per cell compared to the HG in all cell populations under study; however receptor-bound IgG expression decreased significantly 4 h after Dpt extract administration. The elevated expression of FcεRI bound-IgE observed in the SCIT-T0 group could be indicative of an increased FcεRI triggered by the elevated IgE serum levels in allergic patients. On the other hand, 4 h after Dpt extract administration, this condition seems to increase. There also seems to be a negative correlation between the amount of receptor-bound IgE per cell among mDCs and the time under SCIT, which indicates that the treatment has the potential to reduce specific IgE levels in the long term, gradually decreasing IgE-mediated responses.

In our study, SCIT did not result in increased receptor-bound IgG expression. In fact, receptor-bound IgG expression by CD16^+^ monocytes and mDCs was significantly lower in the SCIT-T4 group than in the control group. Besides, all allergic groups displayed less receptor-bound IgG expression in mDCs than the HG. This could be due to the anti-IgG antibody used for IgG detection, which does not allow for differentiation between IgG isotypes and, consequently, is unreliable for the detection of a specific increase in IgG4 or IgG1 levels. Moreover, DCs in the steady state express only low levels of activating Fc receptors for IgG (FcγRs) but express the inhibitory FcγR receptor (FcγRIIB or CD32B) that is involved in maintaining tolerance. Hence we could be targeting IgG bound to this receptor (CD32B) in the healthy group, whereas IgG could be bounded to activating receptors in the case of SCIT groups, triggering completely different immune responses [47]. Furthermore, it has been recently proposed that the role of IgG in the long-term clinical efficiency of SIT probably involves an increase in the avidity of IgG-allergen binding and therefore an increase in its blocking activity, rather than enhanced serum levels [10, 14].

On the other hand, TNFα plays an important role in allergic inflammation by stimulating the production of allergen-specific IgE, chemokines, and Th2-type cytokines [48, 49]. Additionally, monocytes and other immune cells can be stimulated to produce pro-inflammatory cytokines by FcεRI activation and IgE cross-linking. Therefore, TNFα levels are frequently increased in allergic patients [50, 51]. Interestingly, despite the higher amount of receptor-bound IgE per cell observed within SCIT groups, we did not find significant differences in the frequencies of TNFα producing cells among CD16^−^ monocytes. But we found a higher TNFα expression, at the single cell level, in non-SIT allergic participants.

Among mDCs, the non-SIT group displayed higher frequencies of TNFα producing cells. This, in addition to the decrease in circulating mDCs within this group, is in agreement with the activation and recruitment of mDCs to the allergen-exposed tissue [50, 52]. In comparison with the HG, neither the number of circulating mDCs, nor the percentage of TNFα producing mDCs were altered in the SCIT groups.

On the other hand, CD16^+^ monocytes presented higher frequencies of TNFα producing cells in the SCIT groups (both at T0 and T4 evaluations), in comparison to the non-SIT group and HG.

## Conclusions

In summary, our findings demonstrated that SCIT induces significant changes in the homeostasis of peripheral blood monocytes and DC subpopulations, either in number or in their ability to produce pro-inflammatory cytokines. Taken together, these results contribute to a better understanding of the underlying systemic mechanisms induced by SCIT on circulating antigen presenting cells.

## Abbreviations

AR: allergic rhinitis
APC: antigen presenting cells
DCs: dendritic cells
pDCs: plasmacytoid dendritic cells
mDCs: myeloid dendritic cells
Dpt: *Dermatophagoides pteronyssinus*
FcγRs: Fc receptors for IgG
FcεRI: high affinity receptor for IgE
Ig: immunoglobulin
IFNα: interferon α
MHC: major histocompatibility complex
RT: room temperature
SIT: allergen-specific immunotherapy
SCIT: subcutaneous allergen-specific immunotherapy
TNFα: tumor necrosis factor α
